# Association of Polymorphisms in Pharmacogenetic Candidate Genes with Propofol Susceptibility

**DOI:** 10.1038/s41598-017-03229-3

**Published:** 2017-06-13

**Authors:** Qi Zhong, Xiangdong Chen, Yan Zhao, Ru Liu, Shanglong Yao

**Affiliations:** 1 Department of Anesthesiology, Institute of Anesthesiology and Critical Care Medicine, Union Hospital, Tongji Medical College, Huazhong University of Science and Technology, Wuhan, Hubei 430022 China; 2 Department of Anesthesiology, the First Affiliated Hospital of University of South China, Hengyang, Hunan 421000 China

## Abstract

Significant individual susceptibility to intravenous anesthetic propofol exists. The etiology of individual variability in the response to propofol may be influenced by genetic polymorphisms in metabolic and functional pathways. With current pharmacogenetics and modern molecular biology technologies, it is possible to study the influence of genetic polymorphisms on susceptibility to propofol. When inducing general anesthesia with intravenous propofol, high individual susceptibility to propofol was found. Using Sequenom MassARRAY single-nucleotide polymorphism (SNP) genotyping, we identified a mutation (rs6313) in the 5HT2A gene that was correlated to individual susceptibility to propofol effect-site concentration (Cep) and onset time of propofol induction. Carriers of the minor allele (G) of 5HT2A rs6313 required less propofol (20% decrease in Cep) and less time (40% decrease in onset time) to induce anesthesia. Moreover, associations were found between the gamma-aminobutyric acid (GABA) receptor SNP rs2279020 and the SCN9A SNP rs6746030 and the susceptibility of bispectral index (BIS) after propofol-induced anesthesia. In addition, dominant mutations in GABAA1 rs2279020, GABAA2 rs11503014, and CHRM2 rs1824024 were putatively associated with cardiovascular susceptibility to propofol anesthesia. No gene-gene interactions were found through a standardized measure of linkage disequilibrium and a multifactor dimensionality reduction analysis. Our results suggest that genetic polymorphisms related to mechanisms of propofol anesthesia are involved in propofol susceptibility.

## Introduction

Anesthetic and analgesic drugs are widely used in various clinical settings. Although different methods have been developed for the management of applied anesthetics, drug susceptibility is inevitable and difficult to control. Propofol was introduced into clinical practice as a general anesthetic agent in 1977 and has become the agent of choice for rapid intravenous induction. However, susceptibility to propofol anesthesia has been shown to be remarkably variable based on clinical observations of responses of patients of the same ethnic origin, and this variability is reflected in different dose requirements and the amount of required recovery time^1, 2^.

Deep sedation with propofol, which inhibits the stress response^3^, is followed by hypotension; sedation-related complications, or even brain injury^4^, sometimes subsequently deteriorate the outcome of patients. Likewise, light propofol sedation, defined as inadequate anesthesia, would induce hypertension, tachycardia, or patient movement and, more seriously, lead to intraoperative awareness^5^. However, based simply on the traditional dose calculation algorithm of propofol, it is very hard for patients to receive accurate and comfortable anesthesia because of propofol susceptibility. Thus, particular anesthetic measures are needed to provide the most appropriate anesthesia to patients.

The etiology of susceptibility to propofol is complex, involving complicated associations among drugs, biological and psychological factors^6^. More importantly, genetic components could play an essential role in the pathogenesis of susceptibility to propofol^7^.

Studies have demonstrated that individual differences in genetic factors (polymorphisms in selected genes responsible for pharmacokinetics and pharmacodynamics) and nongenetic factors (sex, weight and height) contributed to the variability in dose requirements of propofol^8, 9^. In addition, the anesthesia mechanism of propofol can’t be ignored or even more important for propofol susceptibility, which determined the reaction of loss of consciousness to propofol. Furthermore, mutations in genes involved in the molecular targets and molecular binding sites of propofol may be associated with propofol susceptibility. Therefore, we speculated that genetic polymorphisms in metabolic or functional pathways or receptors might have an influence on individual variability in response to propofol susceptibility.

Previous researches have indicated that the main targets for propofol’s actions are the genes of gamma-aminobutyric acid (GABA) systems; nACR; dopaminergic, serotoninergic, or noradrenergic pathways or associated voltage-dependent ion channels; or enzymes associated with metabolism and mechanisms. Cytochrome P450 family (CYP450), ATP-binding cassette (ABCB1), serine/threonine-protein kinase 3 (TAOK3), family with sequence similarity 53 member B (FAM53B), and the cannabinoid receptor (CNR1) are postulated to be involved in propofol pharmacokinetics; opioid receptors (OPRM1 and OPRD1), β-adrenoceptor (ADRB1), Catechol-O-methyltransferase (COMT), and ligand-gated ion channel (P2RX7) are postulated to be directly or indirectly involved in the pharmacodynamic response to propofol; nitric oxide synthase (NOS3), GABA type A (GABAA) receptor, NMDA receptors (GR1N3A and GR1N2B), Galanin (GAL), fatty acid amide hydrolase (FAAH), 5-hydroxytryptamine receptor (5HT2A), cholinergic receptors (CHRM2 and CHRNA5), dopamine transporters (DAT and DRD2), casein kinase (CSNK1E), calcium channels, potassium channels (KCNS1 and GIRK) and sodium channels (SCN9A) are also likely involved in the action of propofol^7, 10–23^. However, evidence for an association between single-nucleotide polymorphisms (SNPs) in the genes mentioned above and propofol susceptibility in patients is still lacking. Thus, the major objective of this study was to test whether the SNPs in these genes associated with propofol’s metabolism and actions contribute to the variability in individual susceptibility to propofol.

## Results

### Sociodemographic and clinical characteristics of the study subjects

#### Demographic parameters of study groups

In total, 179 of the 192 recruited patients were included in the study. One patient was excluded due to hypertension, and 13 patients were excluded for other reasons (e.g., no continuous data monitoring, no blood provided, or lack of phenotypic data). The key sociodemographic data are summarized in Table 1. All the study participants were Han Chinese. Age, sex and body mass index (BMI) were recorded for all patients. Overall, 83 males and 96 females participated in our study, and the mean age and BMI were 43.33 ± 8.76 years and 23.86 ± 2.38 kg/m^2^, respectively.

**Table 1:** Main sociodemographic data of all study subjects and key clinical characteristics of the patients.

Characteristics	Information
Patients, n	179
Age, years	43.33 ± 8.76
Male/female	83/96
BMI, kg/m^2^	23.86 ± 2.38

#### Clinical characteristics

Under standard total intravenous anesthesia (TIVA), the effect-site concentration (Cep), onset time and total amount of propofol anesthesia, bispectral index (BIS) and hemodynamics of patients were recorded when the patients reached different stages of sedation as determined by the Observer’s Assessment of Alertness/Sedation Scale (OAAS) score.

### Trends in clinical data regarding anesthesia induction and susceptibility to propofol anesthesia

**Figure 1:**
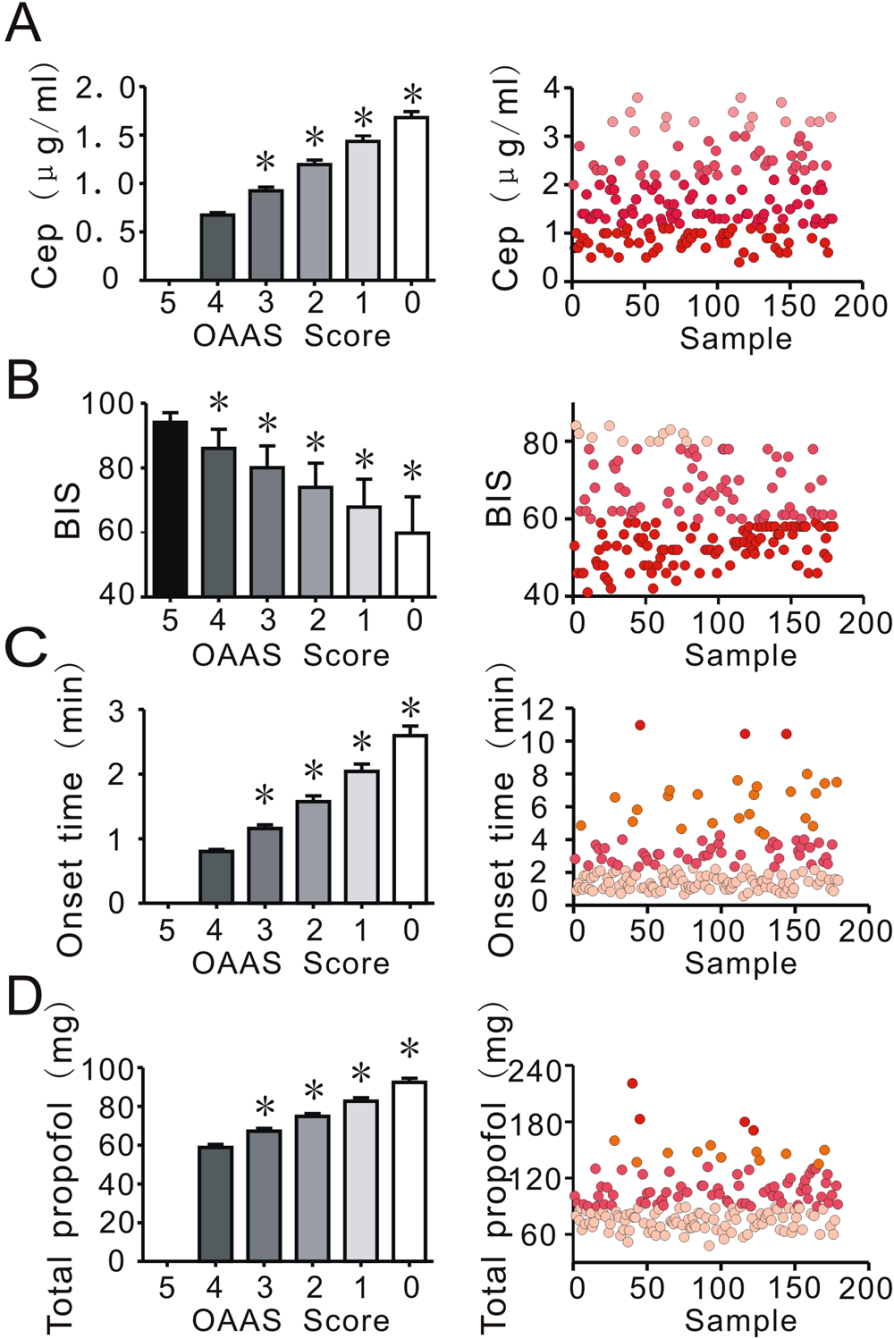
Clinical characteristics of patients during anesthesia induction. Anesthesia was induced with propofol via target-controlled-infusion (TCI) at 4 µg/ml. At OAA/S scores of 5, 4, 3, 2, 1, and 0, the effect-site concentration (Cep), bispectral index (BIS), onset time and total propofol were recorded. (**A**,**B**,**C** and **D**), left, reflect the Cep, BIS, onset time and total propofol recorded at OAA/S scores of 5, 4, 3, 2, 1, and 0. (**A**,**B**,**C** and **D**), right, reflect the distribution of Cep, BIS, onset time and total propofol at the score of 0, when patients lost consciousness. The different colors in Fig. 1, right side, obviously reflect the wide distribution of Cep, BIS, onset time and total propofol values at the score of 0, when patients lost consciousness. Variance between different groups was analyzed by one-way ANOVA. *P < 0.05 vs. Cep and BIS at an OAA/S score of 5 in (**A** and **B**); *P < 0.05 vs. onset time and total propofol at an OAA/S score of 4 in (**C** and **D**).

During anesthesia induction, the intravenous anesthetic propofol induced time- and dose-dependent sedative effects on patients. As shown in Fig. 1, while propofol-induced anesthesia gradually deepened, 0.80 ± 0.41 min, 1.16 ± 0.77 min, 1.58 ± 1.18 min, 2.04 ± 1.54 min, and 2.60 ± 1.99 min (Fig. 1C, left) were required to produce the effects for OAA/S scores 4, 3, 2, 1, 0 respectively. A total amount of propofol required for OAA/S scores of 4, 3, 2, 1, and 0 were 58.90 ± 20.34 mg, 67.21 ± 19.51 mg, 74.91 ± 19.89 mg, 82.75 ± 22.60 mg, and 92.48 ± 27.70 mg, respectively (Fig. 1D, left). The propofol Cep increased from 0.68 ± 0.35 μg/ml, 0.93 ± 0.51 μg/ml, 1.20 ± 0.64 μg/ml, 1.44 ± 0.74 μg/ml, to 1.68 ± 0.83 μg/ml (Fig. 1A, left) and BIS decreased from 86.04 ± 5.94, 80.06 ± 6.72, 73.99 ± 7.50, 67.86 ± 8.67 to 59.79 ± 11.23 (Fig. 1B, left) at OAA/S scores of 4, 3, 2, 1, and 0, respectively. All these clinical data seemed to be well correlated with different levels of sedation, as indicated by different OAA/S scores (Fig. 1, left). However, the results revealed high individual diversity (Fig. 1, right) in dose response under propofol anesthesia. There was a 20-fold difference between the fastest and slowest onset times of propofol anesthesia **(**Fig. 1C, right), which ranged from 0.55 to 10.97 min. In Fig. 1D, right, when the patients lost consciousness, the total propofol infusions were significantly different and ranged from 48 to 221 mg. The Cep of propofol was also highly variable and ranged from 0.40 to 3.80 μg/ml (Fig. 1A, right). The Cep value was 0.4–1.0 μg/ml in 48 patients, 1.0–3.0 μg/kg/min in 115 patients, and 3.0–3.8 μg/ml 16 patients. A nine-fold difference existed between the maximum and minimum Cep values. Figure 1B, right shows that when patients lost consciousness, the BIS varied significantly from 40 to 84, ranging from 40 to 60 in 108 patients, 60 to 80 in 64 patients, and 80 to 84 in 7 patients.

**Figure 2:**
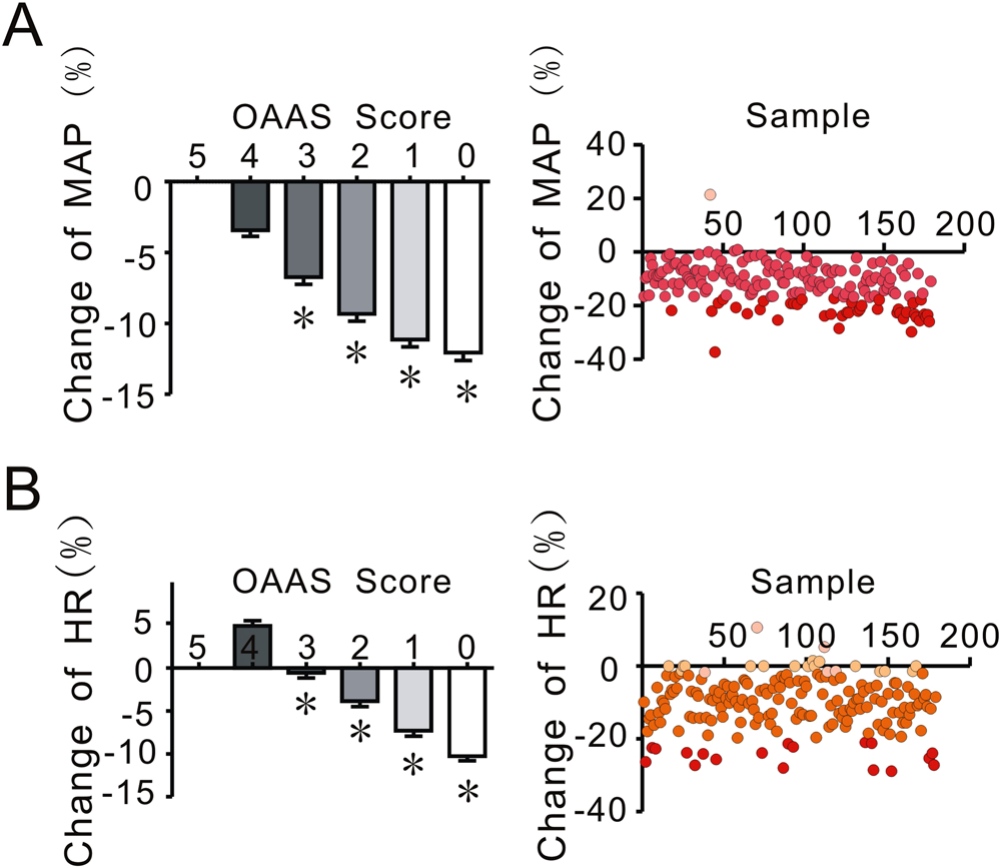
High individual diversity of cardiovascular responses to propofol anesthesia under the condition of unconsciousness in patients. The MAP and HR values of 179 participants at OAA/S scores of 5, 4, 3, 2, 1, and 0 are shown in (**A** and **B**), left. (**A** and **B**), right, show the distribution of MAP and HR changes under the condition of unconsciousness. The different colors in Fig. 1, right side, obviously reflect the wide distribution of MAP and HR changes at the score of 0, when patients lost consciousness. The variance between different groups was analyzed by one-way ANOVA. *P < 0.05 vs. changes in MAP and HR at an OAA/S score of 4 in (**A** and **B**).

Propofol also produced significant effects on hemodynamics, including changes in blood pressure and heart rate during anesthesia induction^24^. As shown in Fig. 2A, with increased sedation from propofol, there was a significant decline in the mean arterial pressure (MAP). The percent changes in MAP were −3.42 ± 5.85%, −7.73 ± 6.94%, −9.32 ± 7.13%, −11.13 ± 7.00%, and −12.05 ± 7.56% at OAA/S scores of 4, 3, 2, 1, and 0 (Fig. 2A, left). As shown in Fig. 2B, except for a slight increase in heart rate (HR) seen at the beginning, propofol induced a significant decline in HR. The HR values changed by 4.92 ± 8.23%, −0.53 ± 8.05%, −3.84 ± 8.68%, −7.29 ± 8.42% and −10.27% ± 7.28% at OAA/S scores of 4, 3, 2, 1, and 0 (Fig. 2B, left), respectively.

As shown in Fig. 2A, right and Fig. 2B, right, the hemodynamic effects of propofol anesthesia also showed significant variability. The MAP values ranged from 60.67 to 98.00 mmHg, and HR ranged from 50 to 98 bpm. The changes in MAP and HR ranged from −37.24% to 21.41% and from −28.87% to 10.61%, respectively, when the patients lost consciousness.

### Genotyping results

#### SNP information

In the present study, 10,919 SNPs (58 SNPs in 179 individuals) were genotyped. The genotype distributions of the 58 SNPs and the minor allele frequency (MAF) of each SNP are shown in Table 2. The test of ADRB1 rs1801253 and DRD3 rs6454674 didn’t show any useful results. In addition, no mutations in ADH4 rs1042363, DRD2 rs9288993 and DRD3 rs6454674 were observed in the patients, all patients carried the major alleles. Except for CNR1 rs324419, ADH4 rs1126671 and COMT rs174696, the frequencies of all the other polymorphisms were in Hardy-Weinberg equilibrium (HWE) (P > 0.05).

**Table 2:** List of the selected candidate genes and polymorphisms.

Symbol	Gene	SNP ID	Alleles	Frequency	HWE P value
OPRM1	Opioid receptor, mu 1	rs1799971	A/G	0.32	1
CYP1A2	Cytochrome P450 family 1 subfamily A member 2	rs2470890	C/T	0.13	0.99
NOS3	nitric oxide synthase 3	rs2070744	C/T	0.08	0.568
GR1N3A	NMDA receptor 3A subunit gene	rs3739722	C/T	0.39	0.852
GR1N2B	NMDA receptor 2B subunit gene	rs3764028	G/T	0.16	0.675
5HT2A	5-Hydroxytryptamine (serotonin) receptor 2A, G protein-coupled	rs6313	A/G	0.49	0.169
GABRB2	Gamma-aminobutyric acid (GABA) A receptor, beta 2	rs2229944	A/G	0.06	0.571
GABRA6	Gamma-aminobutyric acid (GABA) A receptor, alpha 6	rs3219151	C/T	0.32	0.861
GABRA1	Gamma-aminobutyric acid (GABA) A receptor, alpha 1	rs2279020	A/G	0.19	0.971
GABRG2	Gamma-aminobutyric acid (GABA) A receptor, gamma 2	rs211014	A/C	0.45	0.764
		rs211013	A/G	0.29	0.847
GABRA2	Gamma-aminobutyric acid (GABA) A receptor, alpha 2	rs279858	C/T	0.45	0.762
		rs567926	A/G	0.49	0.626
		rs11503014	C/G	0.08	0.578
Calcium channel	Calcium channel				
		rs3846446	A/C	0.38	0.89
KCNS1	Potassium channel alpha subunit	rs734784	C/T	0.20	0.95
		rs2070995	C/T	0.36	0.966
SCN9A	Sodium channel	rs6746030	A/G	0.06	0.982
OPRD1	Opioid receptor, delta 1	rs223686	G/T	0.04	1
ABCB1	ATP-binding cassette, subfamily B, member 1	rs1045642	A/G	0.40	0.97
GAL	Galanin	rs948854	C/T	0.16	0.667
TAOK3	TAO kinase 3	rs795484	C/T	0.49	0.979
FAM53B	Family with sequence similarity 53 member B	rs2629540	C/G	0.27	0.5
CHRNA5	Cholinergic receptor, nicotinic alpha 5	rs16969968	A/G	0.02	1
ADRB1	Adrenoceptor beta 1	rs1801253	None		
		rs737866	C/T	0.30	0.344
COMT	Catechol-O-methyltransferase	rs4680	A/G	0.24	0.618
		rs174696	C/T	0.41	<0.001
ADH4	Alcohol dehydrogenase 4 (class II), pi polypeptide	rs1042363	T	0	1
		rs1229984	C/T	0.29	1
		rs1126671	C/T	0.23	0.01
		rs978437	C/T	0.49	1
CHRM2	Cholinergic receptor, muscarinic 2	rs1455858	C/T	0.45	0.888
		rs1824024	A/C	0.49	0.89
		rs324650	A/T	0.09	1
		rs2283265	A/C	0.41	0.969
DAT	Dopamine transporter	rs1076563	A/C	0.08	1
DRD2	Dopamine receptor D2	rs2587548	C/G	0.09	1
		rs9288993	A	0	1
		rs2654754	A/G	0.01	1
DRD3	Dopamine receptor D3	rs6454674	None		
		rs806368	C/T	0.50	0.474
CNR1	Cannabinoid receptor 1	rs324419	C/T	0.13	<0.001
		rs2295633	A/G	0.20	0.747
FAAH	Fatty acid amide hydrolase	rs2835859	C/T	0.14	0.002
		rs1534891	C/T	0.09	0.807
GIRK	Potassium channel, inwardly rectifying subfamily J, member 3	rs6001093	C/T	0.08	0.574
CSNK1E	Casein kinase 1 epsilon	rs135757	A/G	0.15	0.925
		rs2734849	A/G	0.10	0.988
		rs2708092	A/G	0.46	0.766
ANKK1	Ankyrin repeat and kinase domain containing 1	rs1180012	C/T	0.16	0.72
P2RX7	Ligand-gated ion channel	rs1718125	C/T	0.49	0.482
		rs208293	C/T	0.42	0.971
		rs1718136	A/G	0.13	0.536
		rs7132846	C/T	0.18	0.94
		rs7958311	A/G	0.49	0.477
		rs3751143	A/C	0.17	0.725
		rs208294	C/T	0.37	0.469

HWE, Hardy-Weinberg equilibrium.

#### Correlation between genotype and susceptibility to propofol

The Cep, onset time, BIS, MAP and HR values at OAA/S scores of 0 were selected to evaluate susceptibility to propofol.

Based on the SNPs, the patients were divided into two groups: 1. homozygous for the major allele; and 2. a combination of heterozygous and homozygous for the minor allele. Differences in Cep, onset time, BIS, MAP and HR were analyzed between the two groups. When different patient indexes were compared based on different genotypes, the genes showed significant differences in each index, as shown in Table 3. Unfortunately, the SNPs in the remaining genes, including CYP450, ABCB1, TAOK3, FAM53B, CNR1, OPRM1, OPRD1, ADRB1, COMT, P2RX7, NOS3, GR1N3A, GR1N2B, GAL, FAAH, CHRNA5, DAT, DRD2, CSNK1E, calcium channels, and potassium channels, showed no differences in each index, as shown in Table 4.

**Table 3:** SNPs with detected significant differences in clinical index.

Genotype/alleles	Patients n (frequency)	Cep	MAP (%)	HR (%)	BIS	Time	Total
5HT2A rs6313							
AA	46(0.31)	1.85 ± 0.96	−11.36 ± 7.13	−9.92 ± 7.79	58.96 ± 10.43	187.20 ± 160.80	100.02 ± 35.86
GG + GA	100(0.69)	1.53 ± 0.76*	−11.09 ± 7.24	−10.40 ± 7.12	60.69 ± 12.28	132.00 ± 91.80*	86.51 ± 22.99
GABAA2 rs11503014							
CC	116(0.83)	1.65 ± 0.81	−11.51 ± 6.79	−9.44 ± 6.94	59.84 ± 11.77	149.31 ± 115.68	91.51 ± 28.26
CG	23(0.17)	1.71 ± 1.01	−10.54 ± 9.70	−12.85 ± 8.54*	62.30 ± 10.81	171.05 ± 155.89	92.61 ± 33.35
SCN9A rs6746030							
GG	120(0.88)	1.71 ± 0.87	−11.50 ± 7.73	−10.02 ± 7.41	61.30 ± 10.39	160.01 ± 129.09	93.33 ± 30.07
AA + GA	16(0.12)	1.36 ± 0.59	−10.79 ± 4.06	−9.61 ± 6.85	51.13 ± 15.37*	112.95 ± 58.64	81.19 ± 20.38
GABAA1 rs2279020							
GG	40(0.29)	1.51 ± 0.85	−9.44 ± 5.28	−9.94 ± 6.51	57.15 ± 13.29	139.30 ± 122.11	88.70 ± 27.32
AA + AG	98(0.71)	1.75 ± 0.84	−12.16 ± 7.92*	−10.01 ± 7.68	61.43 ± 10.51*	160.75 ± 123.68	92.97 ± 29.87
CHRM2 rs2283265							
CC	47(0.35)	1.72 ± 0.88	−11.23 ± 6.87	−8.25 ± 7.15	60.92 ± 14.62	154.80 ± 107.56	93.89 ± 33.80
AA + CA	89(0.65)	1.66 ± 0.84	−11.42 ± 7.65	−10.86 ± 7.32*	59.94 ± 10.25	154.26 ± 129.55	90.84 ± 27.47

*P < 0.05 (homozygous carriers of the major allele vs. carriers of the minor allele).

**Table 4:** The SNPs detected no significant difference in clinical index.

Genotype/alleles	Patients n (frequency)	Cep	MAP(%)	HR(%)	BIS	Time	Total
OPRM1 rs1799971							
AA	65(0.47)	1.77 ± 0.85	−11.52 ± 7.47	−9.94 ± 8.23	59.14 ± 12.36	165.44 ± 132.88	92.11 ± 28.01
GG + GA	73(0.53)	1.59 ± 0.84	−11.23 ± 7.27	−10.02 ± 6.50	61.12 ± 10.68	144.82 ± 113.88	91.40 ± 30.26
CYP1A2 rs2470890							
CC	105(0.76)	1.65 ± 0.85	−11.23 ± 7.80	−10.29 ± 7.53	60.70 ± 12.30	151.16 ± 120.69	90.62 ± 30.17
TT + TC	34(0.24)	1.72 ± 0.86	−11.83 ± 5.65	−9.05 ± 6.60	59.24 ± 9.14	161.91 ± 131.54	94.47 ± 25.65
NOS3 rs2070744							
TT	118(0.86)	1.68 ± 0.85	−11.53 ± 7.48	−9.75 ± 7.08	59.75 ± 11.66	155.92 ± 125.31	91.40 ± 29.63
CC + CT	20(0.14)	1.61 ± 0.80	−10.51 ± 6.54	−11.05 ± 8.64	62.00 ± 11.38	141.75 ± 110.23	94.30 ± 26.25
GR1N3A rs3739722							
TT	41(0.38)	1.54 ± 0.81	−10.47 ± 7.04	−10.37 ± 7.20	60.52 ± 14.82	135.14 ± 97.14	88.88 ± 29.66
CC + TC	67(0.62)	1.70 ± 0.84	−12.60 ± 7.61	−9.31 ± 7.69	60.24 ± 9.93	157.64 ± 126.28	92.45 ± 26.25
GR1N2B rs3764028							
TT	107(0.73)	1.62 ± 0.80	80.49 ± 8.39	71.64 ± 9.75	59.47 ± 11.70	146.80 ± 114.53	90.07 ± 25.84
GG + GT	40(0.27)	1.71 ± 0.93	78.39 ± 7.84	70.08 ± 8.37	61.50 ± 11.73	162.39 ± 137.86	94.55 ± 34.60
GABRB2 rs2229944							
GG	121(0.87)	1.66 ± 0.85	−11.45 ± 7.39	−10.26 ± 7.08	60.61 ± 12.08	153.42 ± 125.39	91.60 ± 29.40
GA	18(0.13)	1.76 ± 0.83	−10.62 ± 7.00	−8.41 ± 8.90	58.33 ± 7.05	156.64 ± 108.63	91.56 ± 27.46
GABRA6 rs3219151							
CC	68(0.48)	1.70 ± 0.92	−11.39 ± 7.12	−9.94 ± 7.28	60.13 ± 13.10	164.74 ± 143.60	93.88 ± 33.49
TT + TC	73(0.52)	1.64 ± 0.77	−11.18 ± 7.54	−10.27 ± 7.51	59.95 ± 9.86	143.48 ± 98.38	89.59 ± 23.83
GABRG2 rs211014							
CC	45(0.33)	1.69 ± 0.94	−11.34 ± 6.44	−8.67 ± 7.49	59.69 ± 14.13	163.82 ± 139.37	95.07 ± 30.66
AA + CA	92(0.67)	1.65 ± 0.80	−11.33 ± 7.78	−10.66 ± 7.21	60.35 ± 10.21	148.49 ± 115.39	90.12 ± 28.46
GABRG2 rs211013							
GG	70(0.51)	1.67 ± 0.83	−12.21 ± 8.20	−10.45 ± 7.10	60.03 ± 9.57	152.62 ± 122.48	94.39 ± 31.64
AA + GA	66(0.49)	1.65 ± 0.85	−10.52 ± 6.36	−9.44 ± 7.61	60.64 ± 13.34	151.61 ± 122.53	88.29 ± 25.60
GABRA2 rs279858							
CC	42(0.31)	1.86 ± 0.92	−11.55 ± 7.39	−8.84 ± 6.93	59.36 ± 9.89	180.82 ± 149.05	94.40 ± 32.57
TT + CT	92(0.69)	1.59 ± 0.81	−11.30 ± 7.37	−10.69 ± 7.54	60.92 ± 12.25	143.95 ± 110.47	90.62 ± 27.81
GABRA2 rs567926							
GG	39(0.28)	1.78 ± 0.91	−11.06 ± 7.02	−8.88 ± 7.02	58.23 ± 10.19	170.57 ± 151.59	94.03 ± 31.89
AA + GA	98(0.72)	1.62 ± 0.81	−11.39 ± 7.46	−10.43 ± 7.49	60.98 ± 12.00	146.15 ± 108.89	90.36 ± 27.82
Calcium channel rs3846446							
CC	53(0.38)	1.69 ± 0.94	−11.45 ± 6.93	−10.42 ± 7.19	62.42 ± 10.38	162.25 ± 131.56	94.17 ± 33.88
AA + CA	85(0.62)	1.67 ± 0.79	−11.32 ± 7.63	−9.72 ± 7.46	58.80 ± 11.99	149.72 ± 118.18	90.21 ± 25.81
Potassium channel rs734784							
TT	90(0.64)	1.63 ± 0.88	−11.57 ± 7.69	−9.73 ± 7.18	59.97 ± 10.31	153.76 ± 133.62	91.74 ± 28.98
CC + CT	50(0.36)	1.71 ± 0.78	−10.96 ± 6.67	−10.50 ± 7.58	60.74 ± 13.73	151.40 ± 102.16	91.60 ± 29.44
Potassium channel rs2070995							
CC	55(0.40)	1.64 ± 0.84	−11.20 ± 7.48	−10.06 ± 7.18	61.05 ± 11.46	151.06 ± 120.77	89.11 ± 27.60
TT + TC	83(0.60)	1.70 ± 0.85	−11.48 ± 7.29	−9.93 ± 7.48	59.61 ± 11.56	156.83 ± 125.41	93.47 ± 30.12
OPRD1 rs223686							
GG	32(0.91)	1.70 ± 0.82	−11.78 ± 6.34	−9.81 ± 6.72	58.85 ± 11.23	155.89 ± 124.77	92.45 ± 24.50
GT	3(0.09)	2.47 ± 0.81	−14.71 ± 7.16	−8.05 ± 2.20	54.67 ± 11.37	247.00 ± 151.31	103.33 ± 22.59
ABCB1 rs1045642							
GG	52(0.37)	1.67 ± 0.81	−10.25 ± 8.44	−10.82 ± 7.81	60.54 ± 10.32	152.99 ± 127.58	92.79 ± 32.02
AA + GA	89(0.63)	1.66 ± 0.87	−11.96 ± 6.46	−9.77 ± 7.12	60.08 ± 12.25	152.83 ± 120.00	90.66 ± 27.08
GAL rs948854							
TT	99(0.72)	1.59 ± 0.76	−11.25 ± 6.63	−9.75 ± 7.23	59.83 ± 12.11	141.60 ± 104.22	88.56 ± 24.39
CC + CT	38(0.28)	1.86 ± 1.02	−11.77 ± 9.07	−10.37 ± 7.66	61.24 ± 9.96	186.36 ± 160.38	100.37 ± 38.15
TAOK3 rs795484							
CC	62(0.46)	1.83 ± 0.89	−11.78 ± 6.95	−11.05 ± 7.76	58.81 ± 10.61	174.98 ± 140.60	98.89 ± 35.17
TT + CT	74(0.54)	1.56 ± 0.80	−11.03 ± 7.78	−9.07 ± 6.97	61.08 ± 12.18	139.45 ± 105.87	86.14 ± 21.83
FAM53B rs2629540							
GG	78(0.56)	1.59 ± 0.82	−11.13 ± 6.40	−9.99 ± 7.61	61.30 ± 12.56	142.18 ± 121.10	90.39 ± 30.83
CC + CG	62(0.44)	1.77 ± 0.86	−11.58 ± 8.34	−10.16 ± 6.99	58.89 ± 10.2	167.36 ± 123.98	93.06 ± 26.65
CHRNA5 rs16969968							
GG	133(0.97)	1.65 ± 0.85	−11.44 ± 7.43	−9.68 ± 7.20	60.41 ± 11.62	152.74 ± 125.03	91.47 ± 29.41
AG	4(0.03)	2.23 ± 0.22	−9.90 ± 5.17	−10.82 ± 8.11	53.75 ± 5.44	193.50 ± 131.64	103.75 ± 20.35
ADRB1rs737866							
TT	64(0.44)	1.58 ± 0.84	−11.33 ± 6.81	−9.31 ± 6.33	59.44 ± 12.72	144.57 ± 119.02	91.00 ± 28.99
CC + TC	80(0.56)	1.74 ± 0.84	−11.02 ± 7.73	−10.67 ± 8.03	59.79 ± 10.75	160.46 ± 123.82	92.03 ± 28.46
COMT rs4680							
GG	76(0.56)	1.55 ± 0.81	−12.14 ± 7.08	−10.17 ± 7.75	60.01 ± 12.24	138.67 ± 114.79	87.25 ± 24.16
AA + GA	59(0.44)	1.75 ± 0.83	−10.05 ± 7.55	−9.66 ± 6.92	60.54 ± 10.90	160.45 ± 117.76	96.03 ± 33.52
COMT rs174696							
CC	40(0.42)	1.71 ± 0.88	−11.52 ± 8.32	−9.84 ± 6.60	59.51 ± 9.18	163.11 ± 135.36	96.51 ± 31.83
TT + CT	56(0.58)	1.82 ± 0.82	−10.99 ± 7.65	−10.16 ± 7.68	59.23 ± 10.55	167.26 ± 116.56	93.20 ± 28.06
ADH4 rs1229984							
TT	75(0.51)	1.61 ± 0.83	−11.15 ± 6.56	−8.90 ± 6.86	58.12 ± 12.08	142.16 ± 100.19	89.83 ± 29.29
CC + CT	73(0.49)	1.69 ± 0.84	−11.08 ± 7.96	−11.21 ± 7.55	61.62 ± 10.96	161.09 ± 138.35	92.64 ± 27.38
ADH4 rs1126671							
CC	75(0.54)	1.68 ± 0.86	−11.21 ± 6.68	−9.58 ± 7.50	61.68 ± 13.06	153.80 ± 120.43	92.84 ± 29.80
CT	63(0.46)	1.67 ± 0.83	−11.56 ± 8.10	−10.47 ± 7.17	58.41 ± 9.09	155.40 ± 127.32	90.41 ± 28.47
ADH4 rs978437							
CC	35(0.26)	1.66 ± 0.88	−12.96 ± 7.40	−9.71 ± 8.18	60.80 ± 14.93	149.86 ± 109.25	93.09 ± 32.47
TT + CT	100(0.74)	1.66 ± 0.82	−10.79 ± 7.36	−10.15 ± 7.08	60.07 ± 10.27	152.22 ± 120.61	90.39 ± 27.80
CHRM2 rs1455858							
CC	114(0.83)	1.61 ± 0.88	−10.91 ± 8.37	−10.61 ± 6.97	59.36 ± 10.66	151.68 ± 130.34	87.61 ± 26.50
TT + CT	23(0.17)	1.70 ± 0.83	−11.63 ± 6.87	−9.59 ± 7.50	60.62 ± 11.96	154.99 ± 120.72	93.83 ± 30.33
CHRM2 rs1824024							
CC	37(0.27)	1.72 ± 0.88	−12.56 ± 7.11	−9.70 ± 8.21	60.92 ± 14.62	154.80 ± 107.56	93.89 ± 33.80
AA + CA	100(0.73)	1.66 ± 0.84	−10.94 ± 7.45	−10.13 ± 7.06	59.94 ± 10.25	154.26 ± 129.55	90.84 ± 27.47
CHRM2 rs324650							
TT	115(0.82)	1.68 ± 0.86	−11.68 ± 7.41	−10.49 ± 7.07	60.30 ± 11.91	155.85 ± 125.77	92.76 ± 30.30
AA + AT	25(0.18)	1.62 ± 0.76	−9.70 ± 6.63	−8.14 ± 8.24	59.84 ± 10.10	142.75 ± 108.54	86.24 ± 21.41
DAT rs1076563							
AA	116(0.84)	1.66 ± 0.83	−10.98 ± 7.35	−9.99 ± 7.12	60.82 ± 11.61	150.26 ± 116.57	91.20 ± 29.46
CC + CA	22(0.16)	1.75 ± 0.97	−13.42 ± 7.08	−9.97 ± 8.57	56.86 ± 10.54	177.05 ± 154.68	94.55 ± 27.74
DRD2 rs2587548							
GG	118(0.83)	1.65 ± 0.82	−10.94 ± 7.30	−10.09 ± 7.10	60.84 ± 11.70	149.12 ± 115.94	91.04 ± 29.26
AA + AG	24(0.17)	1.70 ± 0.95	−12.53 ± 7.38	−9.85 ± 8.68	56.33 ± 10.50	172.10 ± 151.61	93.54 ± 27.02
DRD2 rs2654754							
AA	137(0.97)	1.68 ± 0.85	−11.29 ± 7.34	−10.15 ± 7.33	60.15 ± 11.56	153.69 ± 123.68	91.66 ± 29.14
GA	4(0.03)	1.48 ± 0.74	−10.84 ± 6.10	−3.74 ± 1.99	61.50 ± 12.45	123.90 ± 69.73	87.50 ± 18.56
DRD3 rs806368							
CC	39(0.29)	1.82 ± 0.98	−12.99 ± 7.76	−11.00 ± 8.50	60.74 ± 14.25	180.66 ± 153.59	99.33 ± 37.34
TT + TC	96(0.71)	1.61 ± 0.79	−10.77 ± 7.17	−9.49 ± 6.88	59.58 ± 10.00	143.86 ± 109.14	89.23 ± 24.97
CNR1 rs324419							
CC	125(0.83)	1.67 ± 0.84	−11.30 ± 7.25	−9.76 ± 7.17	58.73 ± 11.83	149.88 ± 122.65	92.52 ± 31.35
TT + TC	25(0.17)	1.53 ± 0.83	−10.48 ± 7.16	−12.74 ± 8.25	62.19 ± 10.77	176.57 ± 137.44	95.76 ± 25.59
CNR1 rs2295633							
GG	96(0.64)	1.60 ± 0.82	−11.16 ± 6.98	−9.59 ± 7.68	60.75 ± 12.39	146.74 ± 120.77	89.98 ± 26.53
AA + AG	55(0.36)	1.71 ± 0.85	−11.12 ± 7.63	−11.34 ± 6.80	58.67 ± 10.36	156.80 ± 118.79	92.98 ± 30.79
FAAH rs2835859							
TT	89(0.81)	1.64 ± 0.84	−10.91 ± 6.74	−9.22 ± 7.23	58.73 ± 11.83	149.88 ± 122.64	92.52 ± 31.35
CC + TC	21(0.19)	1.81 ± 0.84	−14.07 ± 6.74	−11.77 ± 6.41	62.19 ± 10.77	176.57 ± 137.44	95.76 ± 25.59
FAAH rs1534891							
CC	114(0.83)	1.67 ± 0.81	−11.58 ± 7.49	−10.25 ± 7.57	61.00 ± 10.69	149.65 ± 111.14	91.51 ± 28.09
TT + CT	23(0.17)	1.68 ± 1.02	−10.47 ± 6.76	−8.28 ± 5.86	56.35 ± 14.72	175.12 ± 173.62	93.43 ± 34.86
GIRK rs6001093							
TT	114(0.85)	1.67 ± 0.82	−11.45 ± 7.55	−10.39 ± 7.61	61.22 ± 10.57	150.83 ± 110.93	92.19 ± 28.06
CT	20(0.15)	1.61 ± 0.98	−10.52 ± 6.10	−8.11 ± 5.38	54.40 ± 14.73	163.05 ± 177.30	87.40 ± 34.47
CSNK1E rs135757							
GG	97(0.70)	1.72 ± 0.83	−11.41 ± 8.02	−10.68 ± 7.67	61.54 ± 10.51	156.28 ± 115.20	93.61 ± 29.95
AA + GA	41(0.30)	1.51 ± 0.86	−11.03 ± 5.23	−8.35 ± 6.26	57.54 ± 13.68	143.12 ± 138.83	85.61 ± 25.82
CSNK1E rs2734849							
AA	117(0.81)	1.65 ± 0.81	−10.92 ± 8.04	−10.06 ± 7.13	60.64 ± 11.66	148.12 ± 114.76	90.91 ± 29.03
GG + GA	28(0.19)	1.63 ± 0.92	−12.70 ± 6.85	−10.66 ± 7.86	57.96 ± 12.11	160.50 ± 142.29	91.61 ± 25.74
CSNK1E rs2708092							
GG	43(0.31)	1.68 ± 0.85	−10.89 ± 7.27	−8.74 ± 6.17	60.16 ± 10.88	152.90 ± 122.18	95.00 ± 35.30
AA + GA	94(0.69)	1.66 ± 0.84	−11.48 ± 7.18	−10.56 ± 7.82	60.21 ± 11.89	153.20 ± 123.26	89.76 ± 25.60
ANKK1 rs1180012							
CC	57(0.42)	1.68 ± 0.82	−11.13 ± 6.11	−10.60 ± 7.37	58.44 ± 10.83	152.78 ± 112.64	95.37 ± 29.73
TT + TC	80(0.58)	1.67 ± 0.87	−11.52 ± 8.18	−9.54 ± 7.37	61.50 ± 11.91	154.46 ± 131.04	88.98 ± 28.71
P2RX7 rs171812							
CC	62(0.45)	1.69 ± 0.80	−11.25 ± 6.09	−10.23 ± 7.58	60.06 ± 11.30	150.52 ± 100.32	93.58 ± 26.84
TT + CT	76(0.55)	1.67 ± 0.89	−11.47 ± 8.26	−9.78 ± 7.18	60.29 ± 11.73	157.80 ± 139.65	90.22 ± 30.95
P2RX7 rs208293							
CC	47(0.33)	1.64 ± 0.76	−10.66 ± 6.50	−10.73 ± 7.61	58.96 ± 11.03	142.91 ± 89.67	93.98 ± 28.73
TT + TC	97(0.67)	1.68 ± 0.89	−11.52 ± 7.74	−9.89 ± 7.16	59.91 ± 11.85	157.56 ± 134.35	90.25 ± 28.57
P2RX7 rs1718136							
AA	113(0.75)	1.64 ± 0.85	−11.21 ± 7.32	−10.32 ± 7.41	59.60 ± 11.98	150.06 ± 124.71	91.54 ± 28.83
GG + GA	38(0.25)	1.67 ± 0.78	−10.96 ± 6.92	−9.94 ± 7.45	61.16 ± 10.91	151.43 ± 105.17	89.68 ± 26.11
P2RX7 rs7132846							
CC	92(0.67)	1.74 ± 0.85	−12.18 ± 6.95	−9.96 ± 7.53	58.88 ± 12.40	161.30 ± 124.25	93.60 ± 30.08
TT + TC	46(0.33)	1.56 ± 0.84	−9.76 ± 7.91	−10.03 ± 7.01	62.80 ± 9.02	140.99 ± 121.17	88.00 ± 27.03
P2RX7 rs7958311							
GG	41(0.30)	1.62 ± 0.80	−11.69 ± 7.00	−9.16 ± 7.11	60.98 ± 13.67	145.90 ± 106.82	89.00 ± 25.48
AA + AG	95(0.70)	1.69 ± 0.87	−11.21 ± 7.55	−10.30 ± 7.44	59.92 ± 10.61	157.30 ± 130.93	92.93 ± 30.84
P2RX7 rs3751143							
AA	101(0.71)	1.73 ± 0.89	−11.18 ± 7.85	−9.78 ± 7.40	60.52 ± 14.82	135.14 ± 97.14	88.88 ± 29.66
CC + CA	41(0.29)	1.52 ± 0.70	−10.94 ± 6.04	−10.34 ± 7.37	60.24 ± 9.93	157.64 ± 126.28	92.45 ± 26.25
P2RX7 rs208294							
TT	49(0.36)	1.64 ± 0.76	−10.79 ± 6.41	−10.66 ± 7.54	59.16 ± 11.45	143.20 ± 89.53	93.96 ± 28.38
CC + CT	86(0.64)	1.68 ± 0.89	−11.65 ± 7.86	−9.59 ± 7.26	60.65 ± 11.63	158.63 ± 138.70	90.20 ± 29.65

As shown in Table 3, the following SNPs predicted susceptibility to propofol anesthesia, as indicated by differences in Cep, onset time, BIS, MAP and HR.

#### 5HT2A rs6313

5-HT (serotonin) is an important amino acid that acts as both a neurotransmitter and a neuromodulator^25^. The 5-HT receptors are located in the central nervous system, and polymorphisms affecting the serotonergic system, such as those in the serotonin transporter (5-HTT) and 5-HT genes, have been linked to regulation of sleep and alertness^26^. In our study, the 31.5% (46/146) of patients who were homozygous for the major allele (AA genotype) showed higher Cep values (1.85 ± 0.96 μg/ml) than the 68.5% (100/146) of patients who were either heterozygous or homozygous for the minor allele (GA + GG) (1.53 ± 0.76 μg/ml) (P = 0.03, df = 1, F = 4.82).

Interestingly, the AA genotype of 5HT2A showed not only higher Cep values but also longer onset times of propofol induction (3.12 ± 2.68 min) than the GA + GG genotypes (2.19 ± 1.53 min) (P = 0.01, df = 1, F = 6.893). Carriers of the minor allele (G) required less propofol and less time for propofol to induce anesthesia.

#### GABA receptors

The GABAA receptor is an important target site of propofol anesthesia^27^. Propofol acts via a wide range of sites, including GABA receptors, and the action of propofol involves the positive modulation of the inhibitory function of the neurotransmitter GABA via GABAA receptors^28^.

#### rs2279020 in GABAA1

In our study, homozygous carriers (28.99%, 40/138) of the major allele (GG) had significantly lower BIS values than those either heterozygous (AG) or homozygous for the minor allele (AA) (57.15 ± 13.29 vs. 61.43 ± 10.51, P = 0.047, df = 1, F = 4.019). This result indicated that carriers of the homozygous major allele (GG) for the GABAA1 receptor (SNP rs2279020) were more susceptible to propofol anesthesia.

In addition, mutation of the GABAA1 receptor (rs2279020) also contributed to the different effects of propofol on blood pressure. The 28.99% (40/138) homozygous carriers of the major allele (GG) had significantly less change in MAP after propofol anesthesia than the 71.01% (98/138) carriers of either heterozygous (AG) or homozygous for the minor allele (AA) (−9.44% ± 5.28% vs. −12.16% ± 7.92, P = 0.048, df = 1, F = 3.967).

#### rs11503014 in GABAA2

In total, homozygous carriers(83.45%,116/139) of the major allele (CC) had significantly lower changes in HR after propofol anesthesia than those either heterozygous (CG) or homozygous for the minor allele (GG) (−9.44% ± 6.94% vs. −12.85% ± 8.54, P = 0.040, df = 1, F = 4.297).

To further analyze the relationship between different GABA receptors and the effects of propofol anesthesia, the SNPs in the GABAA receptor were also evaluated as 2-locus genotype patterns (Table 5) and analyzed for linkage disequilibrium (LD). LD is the non-random association of alleles at different loci; if there is no LD between two alleles at different loci, they are said to be in linkage equilibrium.

**Table 5:** LD comparisons between two genes.

Combinations	Association
SNP1-2	
D′	0.2005
r^2^	0.0043
P	0.8

SNP1: GABRA2 rs11503014. SNP2: GABRA1 rs2279020.

To analyze the LD of GABAA receptor SNP pairs, the standardized LD, as measured by Lewontin’s coefficient (LC: D′ = 0.2005, r^2^: 0.0043, P = 0.8), was estimated using the software 2LD^29^. No strong LD was observed in the GABAA receptor SNP pairs (Table 5).

#### SCN9A rs6746030

Duan *et al*.^30^ demonstrated that SCN9A is related to pain sensitivity and that sodium channels are the target of some anesthetics. Previous studies have provided evidence that clinically relevant concentrations of propofol alter the functions of voltage-dependent sodium channels, which inhibits the synaptic release of glutamate^31^. Regarding the SNP rs6746030 in SCN9A, we recorded a significantly lower BIS value under propofol anesthesia in the 11.76% (16/136) of patients who were either heterozygous or homozygous for the minor allele (GA + AA), compared to the 88.24% (120/136) of patients who were homozygous carriers of the major allele (GG) (51.13 ± 15.37 vs. 61.30 ± 10.39, P = 0.001, df = 1, F = 11.9531). Thus, the minor allele (A) of rs6746030 in SCN9A is involved in susceptibility to propofol anesthesia.

#### CHRM2 rs2283265

The cholinergic muscarinic 2 receptor (CHRM2) is a cholinergic neurotransmitter, and cholinergic stimulation through inhibition of the release of acetylcholinesterase has been shown to have a role in autonomic cardiac control^32^. In our study, propofol produced a significantly less change in HR in the 34.56% (47/136) of homozygous carriers of the major allele (CC) of rs2283265 in CHRM2, compared to the 65.44% (89/136) of patients who were heterozygous or homozygous for the minor allele (CA + AA) (−8.25% ± 7.15 mmHg vs. −10.86% ± 7.32, P = 0.048, df = 1, F = 3.965). Thus, our results indicated that the major allele (C) of CHRM2 may be associated with cardiovascular susceptibility to propofol anesthesia.

To rule out the effects of age, weight and sex in our pharmacogenetic analysis, we further assessed the correlation between the genotype frequencies of the significant SNPs and clinical features of the patients. The results of our statistical analysis are shown in Table 6. There were no significant differences in age or BMI between individuals who were homozygous for the major allele and individuals who were either heterozygous or homozygous for the minor allele for each of the tested SNPs.

**Table 6:** Genotype frequencies of significant SNPs and clinical features of patients.

	Patients, n (frequency)	Mean age, years	Mean BMI
5HT2A rs6313			
AA	46(0.31)	41.92 ± 8.40	24.17 ± 2.44
GG + GA	100(0.69)	43.90 ± 9.01	23.65 ± 2.38
Sodium channel rs6746030			
GG	120(0.88)	42.06 ± 8.62	23.71 ± 2.43
AA + GA	16(0.12)	44.81 ± 9.14	23.85 ± 2.33
GABRA1 rs2279020			
GG	40(0.29)	43.63 ± 8.34	24.07 ± 2.32
AA + AG	98(0.71)	43.06 ± 8.34	23.58 ± 2.43
GABAA rs11503014			
CC	116(0.83)	43.16 ± 8.75	23.75 ± 2.42
CG	23(0.17)	40.04 ± 8.48	23.73 ± 2.46
CHRM2 rs2283265			
CC	47(0.35)	42.72 ± 8.84	23.78 ± 2.35
AA + CA	89(0.65)	42.39 ± 8.67	23.92 ± 2.53

### SNP-SNP interaction reveals moderate synergistic effects

A multifactor dimensionality reduction method (MDR) analysis was used to examine gene-gene interactions and the predictive power of the combined variants^33^. The MDR software provided a number of output parameters for each genetic model. The cross-validation consistency (CVC) score is a measure of the degree of consistency with which the selected model is identified as the best model among all possibilities considered. Testing balanced accuracy (TBA) is a measure of the degree to which the interaction accurately predicts case-control status, with scores between 0.50 (indicating that the model prediction was no better than chance) and 1.00 (indicating a perfect prediction).

Our results showed that rs2279020 in GABAA1 and rs6746030 in SCN9A were involved in susceptibility to propofol anesthesia according to the BIS value; and rs1150314 in GABAA2 and rs2283265 in CHRM2 participated in the hemodynamic response to propofol anesthesia according to the HR results. We performed an MDR analysis to examine the significant SNP-SNP interactions. Compared to rs6746030 in SCN9A (TBA 0.5443, CVC 6, P = 0.9325) alone, the interaction between rs2279020 in GABAA1 and rs6746030 in SCN9A (TBA 0.5723, CVC 10, P = 0.6142) was highly significant. Likewise, compared to rs2283265 in CHRM2 (TBA 0.525, CVC 6, P = 0.4850), the interaction between rs2279020 in GABAA1 and rs6746030 in SCN9A (TBA 0.650, CVC 10, P = 0.2617) was also highly significant. As shown in Table 7, despite the high TBA and CVC of rs2279020 in combination with rs6746030 compared to that of rs6746030 alone, there was no interaction between these two SNPs, indicating that the GABA receptor and sodium channels did not produce synergistic effects on propofol susceptibility. Similarly, the GABA receptor and CHRM2 did not produce synergistic effects on cardiovascular susceptibility to propofol anesthesia.

**Table 7:** MDR interaction analysis between SNPs.

Type	MDR models	TBA ^a^	CVC ^b^	P value ^c^
BIS	rs6746030	0.5443	6	0.9325
	rs2279020-rs6746030	0.5723	10	0.6142
HR	rs2283265	0.525	6	0.4850
	rs1150314-rs2283265	0.650	10	0.2617

^a^Testing Balance Accuracy. ^b^Cross-Validation Consistency. ^c^P-values as calculated after 1000 permutations.

## Discussion

In this study, we systemically investigated the roles of multiple polymorphisms in susceptibility to propofol anesthesia. Our results demonstrated that there were significant differences in individual susceptibility to propofol anesthesia and that different patients required different times and different Cep to produce different BIS values and different hemodynamic responses at the same level of propofol anesthesia. Several important molecular targets, including the 5HT receptor, GABA receptor and sodium channels, were shown to contribute to susceptibility to propofol anesthesia.

Previous experimental and clinical studies have demonstrated that individual susceptibility to propofol anesthesia is dependent on factors governing drug susceptibility and drug disposition^34^. Moreover, great inter-patient variability exists in the dose of propofol required to achieve a BIS value <70^1^. Although potential susceptibility to propofol has been reported in vitro^35^, traditional methods of clinical anesthesia render it difficult to judge susceptibility to propofol^4, 5^. It is difficult to maintain appropriate propofol anesthesia because of individual susceptibility.

Target controlled infusion (TCI) systems were used to precisely deliver propofol in our study. The device provides precise control of the propofol level (the target concentration) at the Cep. BIS is used to measure the depth of anesthesia. In our study, when the patients lost consciousness, their BIS scores were approximately 60, which is similar to scores recorded after induction by propofol in other studies^36^. After the loss of patient consciousness, the Cep, onset time and BIS of propofol anesthesia can be used to evaluate susceptibility to propofol. In our study, there was a 20-fold difference between the fastest and slowest onset times for anesthesia induction when patients were anesthetized with propofol to reach a steady unconscious state. The Cep values of propofol were highly variable, showing a 9-fold difference between the most and least sensitive patients. The BIS of propofol anesthesia in patients also ranged from 40 to 84 at the same level of sedation.

Genetic components play an essential role in susceptibility to propofol. Early studies evaluating susceptibility to propofol focused on only the CYP450 gene, which is involved in the metabolism of propofol. Mourão *et al*.^37^ found that a polymorphism in the CYP2B6 gene (rs3745274) affected susceptibility to propofol anesthesia, showing that the total propofol doses based on the GG or GT/TT genotypes were 151.5 mg and 129.3 mg. Mastrogianni *et al*.^38^ also indicated that the c.516G> T polymorphism in CYP2B6 was associated with high blood propofol concentrations. In addition, Lian *et al*.^39^ demonstrated that the impact of a polymorphism in CYP2C9 contributed to susceptibility to propofol. All these studies indicated that enzymes involved in the metabolism of propofol affect susceptibility to propofol and that the total propofol consumption can be disrupted by other anesthetics used during surgery. Our study didn’t only pay attention to the genes involved in the pharmacokinetics and pharmacodynamics of propofol anesthesia, but also aimed to test whether the genes associated with the mechanism of action of propofol affect susceptibility to propofol. Unfortunately, unlike the above studies demonstrating that the metabolism of propofol influenced susceptibility to propofol, our study showed that no SNPs involved in the metabolism of propofol were associated with susceptibility to propofol anesthesia. This trend may result from our research design, which was focused only on propofol-induced anesthesia to avoid interference by other anesthetics. The short time may have limited the functions of SNPs involved in the metabolism of propofol.

In this study, using a candidate gene approach, significant associations between the pharmacogenetic variants of these genes and susceptibility to propofol were identified. We found that polymorphisms in the 5-HT receptor, GABA receptor and sodium channel were associated with susceptibility to propofol anesthesia. Moreover, due to the observation of wide ranges of HR and MAP values, differences in HR or MAP between patients were compared based on the SNP results. Interestingly, polymorphisms in GABA receptors and the cholinergic receptor were associated with hemodynamic responses to propofol.

The 5-HT receptor is involved in the regulation of brain activity and function, and it participates in sensory processing, sleep and alertness^40^. Highly effective modulation is completed by releasing 5-HT to targeted areas, in which several pre- and postsynaptic receptors are implicated^20^. Networks are important for developing cortical neural circuits^41^, and some areas in the networks are associated with anesthesia; these areas of the network include the sleep-promoting ventrolateral preoptic area (VLPO) neurons that contribute to anesthetic hypnosis^42^. Notably, Okamoto *et al*.^43^ confirmed that 5HT2A was involved in antinociceptive actions.

We found that carriers of the minor allele (G) of rs6313 showed lower Cep values and shorter onset times for propofol anesthesia than patients who were homozygous for the major allele (AA). Several reports have suggested that anesthetic doses of propofol increase serotonergic activity^44^, and propofol may facilitate 5-HT release in the brain by increasing serotonergic metabolism. The variation of rs1636 in 5-HT2A from A to G may change the metabolism of 5-HT under propofol anesthesia, and the release of 5-HT increases in carriers of the minor allele of the rs6313 SNP in the 5-HT2A receptor compared with patients who are homozygous for the major allele (AA). Thus, carriers of the minor allele of rs6313 may show stronger activation of the sleep-promoting VLPO neurons that contribute to anesthetic hypnosis. This result may significantly contribute to elucidating the role of 5HT2A in susceptibility to propofol anesthesia.

GABA is the primary inhibitory neurotransmitter in the mammalian brain, and GABA-mediated transmission is involved in adjusting three interactive states: sleep, anesthesia, and pain^45, 46^. GABA regulates these processes by activating GABAA receptors and GABAB receptors. GABAA receptors permit chloride ions to access the center of the pentamer and the binding sites of GABA and modulatory drugs; these sites include binding sites for various anesthetics^47, 48^. Binding to the agonist sites of GABAA is believed to contribute to the hypnotic effects of propofol.

Based on our study, the G-to-A mutation in rs2279020 in GABAA1 may change the pharmacological properties of the receptor by varying the composition and arrangement of subunits^49^. Under propofol anesthesia, the minor A allele of rs2279020 in GABAA1 may induce a stronger inhibition in the brain, as shown by the higher BIS in those patients after the loss of consciousness. This result significantly supports the role of GABAA1 in susceptibility to propofol anesthesia.

Sodium channels are an important regulator of neuronal action potentials, and previous studies have provided evidence that clinically relevant concentrations of propofol alter the functions of voltage-dependent sodium channels, thereby inhibiting the synaptic release of glutamate^31^. Thus, sodium channels were considered an important target of anesthetics^50^. The SCN9A gene, which encodes the Nav1.7 voltage-gated sodium channel, is associated with different abnormal pathophysiological conditions, such as human pain sensitivity^51, 52^. In addition, recent studies have indicated that propofol can act directly on sodium channels to modulate intrinsic ionic conductance^53^.

In our study, the significant role of SCN9A in susceptibility to propofol was confirmed. In patients who were heterozygous or homozygous for the minor allele (A) of the rs6746030 SNP in SCN9A, we recorded significantly lower BIS values. Previous reports have suggested that anesthetic doses of propofol inhibit glutamate release; the decrease in the excitatory neurotransmitter may remodel brain activity, followed by a state of anesthesia. The variation in rs6746030 in SCN9A from G to A may change the function of the sodium channel; the changes in glutamate release and intrinsic ionic conductance resulted in greater susceptibility to propofol, which induced significantly lower BIS values after propofol-induced loss of consciousness. This result may significantly contribute to elucidating the role of SCN9A in susceptibility to propofol anesthesia.

Previous studies have demonstrated that propofol produces inhibitory effects on the cardiovascular system, including reductions in blood pressure and HR^54^; these effects were also found in our current results. In the present study, MAP tended to decrease following anesthesia induced by the TCI of propofol. HR tended to increase at first, followed by a decrease. Our study also showed significant variability in the hemodynamic effects induced by propofol anesthesia, with MAP values ranging from 60.67 to 98.00 mmHg and HRs ranging from 50 to 98 bpm when the patients lost consciousness.

The effects of propofol on cardiovascular systems include depressing the activity of the cardiovascular nervous center, reducing cardiac function and influencing the vascular system^55^. Because the initial purpose of our study was to explore polymorphisms in genes involved in the molecular mechanism of propofol anesthesia, this study analyzed only the relationship between polymorphisms in central nervous system targets and hemodynamic susceptibility to propofol. Interestingly, we found that polymorphisms in the GABAA receptor and the CHRM2 receptor contributed to hemodynamic susceptibility to propofol.

Our current study showed that propofol significantly lowered the HR upon loss of consciousness in patients who were carriers of the minor allele G of rs11503014 in GABAA2 compared with that in patients without the G allele. Moreover, the variation in the C-to-A polymorphism of rs2283265 in CHRM2 resulted in lower HR values. The variation from G to A of rs2279020 in GABAA1 resulted in lower MAP after propofol anesthesia. These results were somewhat surprising because little is known about the effects of anesthetics on the central regulation of the cardiovascular system.

Actually, several targets in the central nervous system play important roles in the regulation of the cardiovascular system. GABAAs play an important role in hemodynamic balance: accumulating evidence suggests that GABAergic inhibition in the rostral ventrolateral medulla (RVLM) and hypothalamic paraventricular nucleus (PVN) significantly contributes to the sympatho-excitation associated with cardiovascular-related disorders, such as hypertension and heart failure^56^. In our study, the polymorphisms rs2279020 in GABAA1 and rs11503014 in GABAA2 may have changed the structure of the GABA receptor, thereby influencing the binding of propofol. These changes may have affected the control of cardiovascular and sympathetic functions in the RVLM and PVN.

The CHRM2 receptor is a cholinergic neurotransmitter, and cholinergic stimulation via the inhibition of acetylcholinesterase release has demonstrated the role of the cholinergic system in autonomic cardiac control^32^. Evidence from animal models and clinical trials suggests that modulating cholinergic activity and restoring sympathovagal balance has salutary effects on hemodynamics^57^. Gamou *et al*.^58^ demonstrated that a microinjection of propofol into the perifornical area of rats induced a decrease in the cortical release of acetylcholine. We speculate that CHRM2 may be a target of propofol and that the activation of CHRM2 by propofol may decrease the HR via decreasing release of acetylcholine. Variations in rs2283265 in CHRM2 may change the structure and function of CHRM2, resulting in a decrease in the inhibitory effects of propofol on HR.

In summary, our study showed that the GABAA receptor and cholinergic receptors contributed to cardiovascular susceptibility to propofol anesthesia. Interestingly, only the GABAA1 receptor was implicated in genetic susceptibility to both propofol-induced anesthesia and the hemodynamic response to propofol. This suggested that the intravenous anesthetic propofol most likely produced anesthesia and cardiovascular action by different mechanisms. These results indicated that evaluating the anesthesia level of propofol through a simple hemodynamic response is insufficient.

To investigate whether other factors influenced the differences in Cep, onset time, BIS and hemodynamics, age and BMI were compared between individuals who were homozygous for the major allele and individuals who were heterozygous or homozygous for the minor allele for each of the tested SNPs. As shown in Table 6, there were no significant differences in age or BMI between the two groups, which demonstrate that the differences in Cep, onset time, BIS and hemodynamics were likely not influenced by other factors.

Based on the LD and MDR analyses, none of the genes were found to interact with each other. This result was somewhat disappointing because we originally expected that these molecular targets might interact with each other to synergistically contribute to both anesthesia and the hemodynamic response to propofol. This prediction was based on recent studies of the networks in the central nervous system related to anesthetic actions^59^.

The current study demonstrated that patients with different pharmacogenetic polymorphisms had individualized susceptibility to propofol anesthesia. Thus, based simply on the traditional dose calculation algorithm for propofol, patients with the minor allele (G) for rs6313 in 5HT2A, homozygous carriers of the major allele (GG) of rs2279020 in GABAA1, and carriers of the minor allele (A) of rs6746030 in SCN9A may be more likely to experience an overdose of propofol. This result may allow us to implement a preoperative genetic screening to identify individuals with a high risk of experiencing a propofol-induced anesthetic overdose. In addition, propofol induced lower HR in patients carrying the minor allele (G) of rs11503014 in GABAA2 and the minor allele (A) of rs2283265 in CHRM2 as well as lower MAP in carriers of the minor allele (A) of rs2279020 in GABAA1. This outcome should remind us to provide better preoperative screening for individuals at high risk of cardiovascular susceptibility to propofol anesthesia.

The results of this study also demonstrated that propofol susceptibility is not determined by a single factor and that some genes associated with the mechanisms of propofol action are involved in propofol susceptibility. There are some limitations in our study. The study sample size was relatively small; we did not measure plasma concentrations of propofol but, rather, forecasted concentrations of propofol^8^; the blood- and effect-site concentrations may not have been consistent; the genes we selected were based only on existing polymorphisms, while many genes with undiscovered polymorphisms may have effects on the action of propofol. The findings of genetic susceptibility targets in this study may contribute to the understanding of the neurobiological mechanism underlying the propofol susceptibility and aid in the development of novel anesthetic options.

## Materials and Methods

### Study participants

For the current candidate gene association study, 192 individuals undergoing thyroid gland resection were recruited at the operating theater of the Union Hospital of Tongji Medical College, Huazhong University of Science and Technology, China. The following inclusion criteria were applied: (1) written informed consent; (2) patients were undergoing elective surgery, were 25–55 years of age, had a BMI of 20–30 kg/m^2^, and were ASA I or II; (3) no history of surgery; and (4) no history of drug addiction. Patients were not included if they met one of the following exclusion criteria: (1) allergies or a history of drug dependence; (2) pregnant or lactating; and (3) ASA III or IV. These criteria were confirmed in a structured personal interview and by a self-designed questionnaire for data collection. All the interviews were conducted by the same physician.

### Assessment of sedation by propofol anesthesia

The state of sedation was assessed using the BIS^60^ and OAA/S scale (score 5 = awake and responds readily to name spoken in normal tone; 4 = lethargic response to name in normal tone; 3 = response only after name is called loudly or repeatedly; 2 = response only after name is called loudly and after mild shaking; 1 = does not respond when name is called and after mild shaking)^61^.

### Study procedures

Patients did not receive medication prior to the procedure. Standard monitoring procedures, including non-invasive arterial pressure (NIAP), electrocardiography, pulse oximetry and BIS measurements, were conducted upon patient arrival in the operating room. The MAP, HR and BIS values were recorded.

Anesthesia was induced with propofol via TCI at 4 µg/ml with the pharmacokinetic and pharmacodynamic (PK-PD) model introduced by Schnider *et al*.^8^. Another investigator then recorded the OAA/S score every 30 s until the patient was sedated to an OAA/S score of 0. At OAA/S scores of 4, 3, 2, 1, and 0, the Cep, onset time, BIS, MAP and HR values were recorded.

Skilled anesthetists inserted a tracheal catheter for airway management, and mechanical ventilation was started with intravenous remifentanil and cis-atracurium injections after the patient lost consciousness. Anesthesia was maintained with propofol TCI (3 μg/ml), and the dose of remifentanil was maintained to ensure that the patient maintained stable vital signs.

### Assessment of susceptibility to propofol

Cep, onset time, and BIS at an OAA/S score of 0 were selected to demonstrate susceptibility to propofol.

### Assessment of cardiovascular susceptibility to propofol

MAP and HR at an OAA/S score of 0 were selected to demonstrate cardiovascular susceptibility to propofol.

### Genotyping

#### Selection of polymorphic loci

This was a candidate gene association study. Based on knowledge of metabolic pathways, transporters, targets and the mechanism of action of propofol, the genes and polymorphic loci were selected after an extensive literature study. The main criterion for the selection of genes and polymorphic loci was evidence speculating or confirming a functional correlation. In total, 58 SNPs located in 35 different genes were selected (Table 2). Five of the 35 investigated genes were postulated to be involved in propofol pharmacokinetics (CYP1A2, ABCB1, TAOK3, FAM53B, and ADH4), ten genes were postulated to be directly or indirectly involved in the pharmacodynamics of the response to propofol (OPRM1, GR1N3A, GR1N2B, OPRD1, GAL, ADRB1, COMT, CNR1, P2RX7, and FAAH), and twenty genes are known to participate in the mechanism of action of propofol (NOS3, GABAB2, GABAA6, GABAA1, GABAG2, GABAA2, GABA, 5HT2A, 5-HTTLPR, CHRNA5, CHRM2, DAT, DRD2, DRD3, CSNK1E, ANKK1, calcium channel, potassium channel, GIRK, and sodium channel).

#### DNA sample collection and DNA extraction

One milliliter of arterial blood was sampled from the femoral artery of each patient after anesthesia induction. Fresh blood was stored at −80 °C and was subsequently extracted using a standard phenol-chloroform procedure. Genotypes were determined using a Sequenom MassARRAY SNP genotyping system, which was based on detection through MALDI-TOF MS (Sequenom Inc., San Diego, CA, USA).

### Statistical analysis

Statistical analyses were performed with SPSS 20.0 (SPSS Inc., Chicago, IL, USA). All values were expressed as the means ± SD. Differences in clinical characteristics among different OAA/S scores were assessed using ANOVA and Wilcoxon signed-rank tests. Furthermore, Chi-square testing was applied to assess the HWE of each of the tested SNPs, and P < 0.05 indicated deviation from equilibrium. The genotypes of each tested SNP were divided into two groups: homozygous for the major allele and a combination of heterozygous and homozygous for the minor allele. In this study, dichotomization of genotypes was necessary because the number of subjects with the minor allele for some SNPs was <5. Furthermore, the statistical power would not have been sufficiently high (<60%) if the genotypes were trichotomized and comparisons were made between homozygotes for the major allele and homozygotes for the minor allele for some SNPs. For data with a normal distribution, differences in Cep, MAP, HR, induction time, total infusion and BIS among SNPs were determined using an ANOVA test. Data with an abnormal distribution were compared using a Wilcoxon signed-rank test, and a P value of <0.05 was considered significant. The standardized measure of LD was calculated with the 2LD and SPSS 20.0 software programs for each pair of markers and was indicated by a coefficient of LD (D′) and *r* ^2^. D′ >0.7 was considered eligible for LD.

The MDR method, which is described in detail elsewhere, was used to examine gene-gene interactions and the predictive power of combined variants. MDR was used to assess all possible genetic models by reducing the dimensionality of the genotype determinants and providing the best genetic model for predicting outcomes. The MDR software provided a number of output parameters for each genetic model. The cross-validation consistency score was a measure of the degree of consistency with which the selected model was identified as the best model among all possibilities considered. Testing balanced accuracy is a measure of the degree to which the interaction accurately predicts case-control status, with scores between 0.50 (indicating that the model prediction was no better than chance) and 1.00 (indicating a perfect prediction).

### Study approval

**Figure 3:**
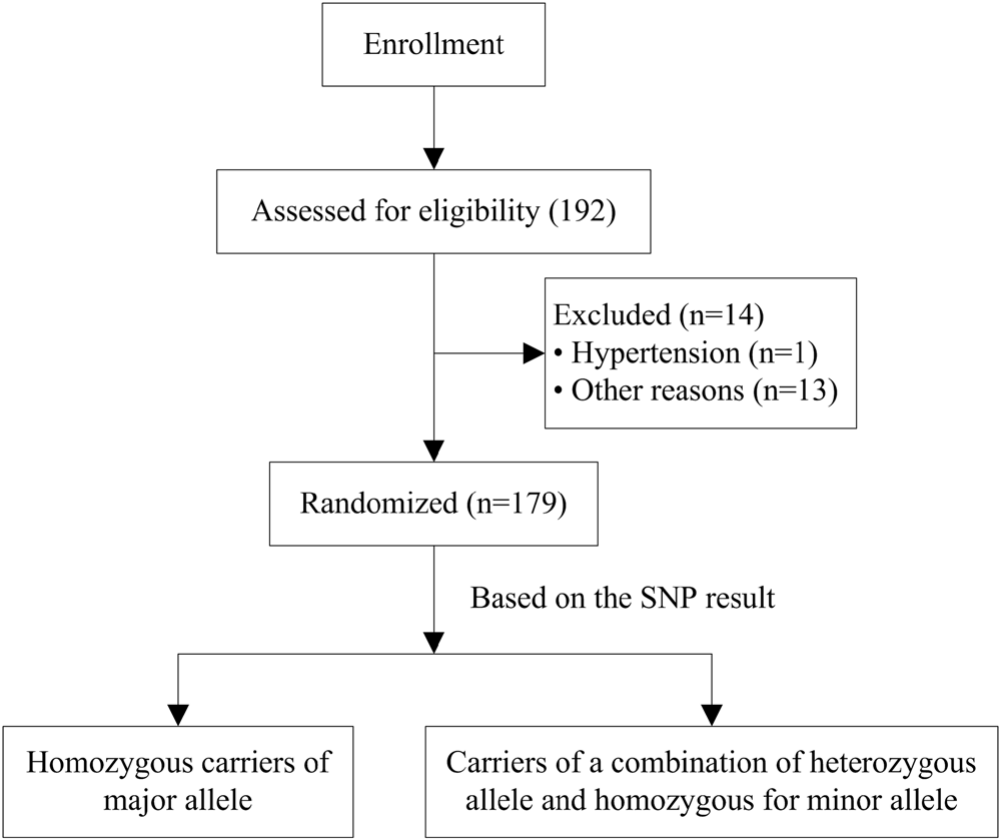
Flow chart. Among the 192 participants enrolled in our study, one participant showed hypertension at the induction of anesthesia, and the remaining 13 early terminations were due to other reasons (no continuous monitoring data, no blood provided, or lack of phenotypic data). Based on the SNP results, the patients were divided into two groups. By comparing the clinical characteristics between the two groups, susceptibility to propofol was determined.

The present study was approved by the Union Hospital, Tongji Medical College, Huazhong University of Science and Technology, China. All methods were performed in accordance with the relevant guidelines and regulations. All participants signed an informed consent after receiving a complete description of the study and were given the chance to discuss any questions or issues. The procedures performed in the research are depicted in Fig. 3.
